# Supplementary material for: Identification of Freezing-Responsive microRNAs and Their Targets in Chinese Jujube by Small RNA and Degradome Sequencing
Source: Int J Mol Sci. 2026 Sep 4;27(17):7912. doi: 10.3390/ijms27177912 (PMC13566890; doi:10.3390/ijms27177912)
Supplement: Supplementary file 1 [file ijms-27-07912-s001.zip › Supplementary Material S2/novel_miR_94.pdf]

Provisional ID	:	novel_miR_94
Score total	:	2.3
Score for star read(s)	:	-1.3
Score for read counts	:	0
Score for mfe	:	2
Score for randfold	:	1.6
Score for cons. seed	:	
Total read count	:	2469
Mature read count	:	1602
Loop read count	:	2
Star read count	:	865

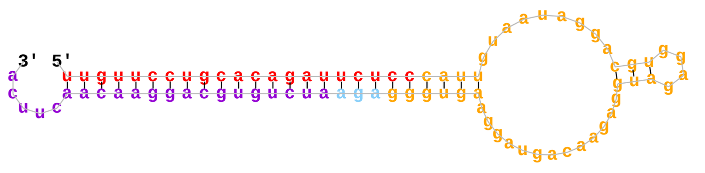

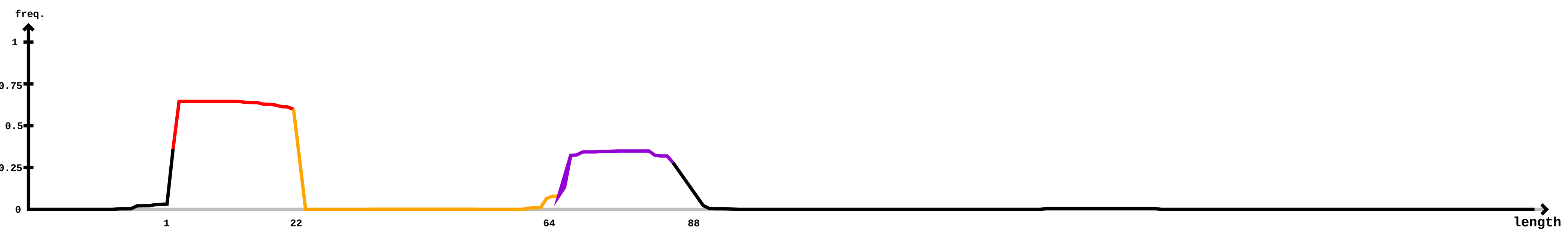

Mature Star

```
5'- nnnnnnnnnnnnuuguugaaguuguuccugcacagauucucccauuguaauaggacguggagauggagaacaguaggaagugggagaaucugugcaggaacaacuucaacaauaucaacaagaucaacagggagcaaugggagaaaaugugcacgaacaauaucaacagaaucaacagggaagaagcacugcuccaccugguguuugccuuuccuauguaacuccaaaugccaucuuaucuauaacgca -3' obs
    nnnnnnnnnnnnuuguugaaguuguuccugcacagauucucccauuguaauaggacguggagauggagaacaguaggaagugggagaaucugugcaggaacaacuucaacaauaucaacaagaucaacagggagcaaugggagaaaaugugcacgaacaauaucaacagaaucaacagggaagaagcacugcuccaccugguguuugccuuuccuauguaacuccaaaugccaucuuaucuauaacgca     exp
    ............((((((((((((((((((((((((((((((((.........(((....)))............)))))))))))))))))))))))))))))))).......((((....(((..(((...((((....(((......)))................(((((((..(((..((.((....)).)).))))))))))......))))...)))..))).))))........ reads mm sample
    ............uuguugaaguuguuccugca......................................................................................................................................................................................................................... 1 0 LT-3
    ............uuguugaaguuguuccugcac........................................................................................................................................................................................................................ 2 0 LT-1
    ............uuguugaaguuguuccugcac........................................................................................................................................................................................................................ 4 0 JCK-3
    ............uuguugaaguuguuccugcac........................................................................................................................................................................................................................ 1 0 LT-3
    .............uguugaaguuguuccugcaca....................................................................................................................................................................................................................... 1 0 LT-1
    ...............uugaaguuguuccugcac........................................................................................................................................................................................................................ 1 0 JCK-3
    ...............uugaaguuguuccugcac........................................................................................................................................................................................................................ 1 0 LT-3
    ...............uugaaguuguuccugcac........................................................................................................................................................................................................................ 3 0 JCK-1
    ...............uugaaguuguuccugcac........................................................................................................................................................................................................................ 3 0 LT-1
    ...............uugaaguuguuccugcacag...................................................................................................................................................................................................................... 1 0 JCK-2
    ...............uugaaguuguuccugcacag...................................................................................................................................................................................................................... 1 0 JCK-3
    ...............uugaaguuguuccugcacag...................................................................................................................................................................................................................... 1 0 LT-3
    ...............uugaaguuguuccugcacaga..................................................................................................................................................................................................................... 1 0 LT-3
    ...............uugaaguuguuccugcacaga..................................................................................................................................................................................................................... 2 0 LT-2
    ...............uugaaguuguuccugcacaga..................................................................................................................................................................................................................... 5 0 JCK-3
    ...............uugaaguuguuccugcacaga..................................................................................................................................................................................................................... 5 0 JCK-1
    ...............uugaaguuguuccugcacaga..................................................................................................................................................................................................................... 10 0 JCK-2
    ...............uugaaguuguuccugcacagauu................................................................................................................................................................................................................... 7 0 JT-3
    ...............uugaaguuguuccugcacagauu................................................................................................................................................................................................................... 3 0 JT-2
    ...............uugaaguuguuccugcacagauC................................................................................................................................................................................................................... 1 1 LT-1
    ................ugaaguuguuccugcacaga..................................................................................................................................................................................................................... 1 0 JCK-3
    ................ugaaguuguuccugcacagau.................................................................................................................................................................................................................... 1 0 JCK-3
    ..................aaguuguuccugcacagauuc.................................................................................................................................................................................................................. 5 0 JCK-2
    ..................aaguuguuccugcacagauuc.................................................................................................................................................................................................................. 8 0 JCK-3
    ..................aaguuguuccugcacagauuc.................................................................................................................................................................................................................. 3 0 JCK-1
    ...................aguuguuccugcacagauu................................................................................................................................................................................................................... 1 0 LT-2
    ...................aguuguuccugcacagauuc.................................................................................................................................................................................................................. 1 0 JCK-2
    ...................aguuguuccugcacagauuc.................................................................................................................................................................................................................. 2 0 JCK-3
    ...................aguuguuccugcacagauucu................................................................................................................................................................................................................. 1 0 JCK-2
    ....................Cuuguuccugcacagauucucc............................................................................................................................................................................................................... 3 1 JCK-2
    ....................Cuuguuccugcacagauucucc............................................................................................................................................................................................................... 1 1 JCK-1
    ....................Cuuguuccugcacagauucucc............................................................................................................................................................................................................... 1 1 JCK-3
    .....................uuguuccugcacagauuc.................................................................................................................................................................................................................. 1 0 JCK-3
```

Mature **Star**

```
nnnnnnnnnnnnnuuguugaaguuguuccugcacagauucucccauuguaauaggacguggagauggagaacaguaggaagugggagaaucugugcaggaacaacuucaacaauaucaacaagaucaacagggagcaaugggagaaaaugugcacgaacaauaucaacagaaucaacagggaagaagcacugcuccaccugguguuugccuuuccuauguaacuccaaaugccaucuuaucuauaacgca
......................uuguuccugcacagauuU..................................................................................................................................................................................................   2   1   JCK-1
......................uuguuccugcacagauuc..................................................................................................................................................................................................   2   0   JCK-1
......................uuguuccugcacagauuU..................................................................................................................................................................................................   1   1   JCK-3
......................uuguuccugcacagauucu.................................................................................................................................................................................................   1   0   JCK-2
......................uuguuccugcacagauucu.................................................................................................................................................................................................   1   0   JCK-1
......................uuguuccugcacagauucuc................................................................................................................................................................................................   2   0   JCK-3
......................uuguuccugcacagauucuc................................................................................................................................................................................................   4   0   LT-2
......................uuguuccugcacagauucuc................................................................................................................................................................................................   1   0   JCK-1
......................uuguuccugcacagauucuc................................................................................................................................................................................................   3   0   JCK-2
......................uuguuccugcacagauucucc...............................................................................................................................................................................................   52  0   LT-3
......................uuguuccugcaNagauucucc...............................................................................................................................................................................................   1   1   JCK-1
......................uuguuccugcacagauucuUc...............................................................................................................................................................................................   1   1   JCK-2
......................uuguuccugcacCgauucucc...............................................................................................................................................................................................   1   1   JCK-3
......................uuUuuccugcacagauucucc...............................................................................................................................................................................................   1   1   JCK-2
......................uuguuccugcacagauucucc...............................................................................................................................................................................................   291 0   JCK-2
......................uuguuccugcacagauucucc...............................................................................................................................................................................................   174 0   JCK-1
......................uuguuccugcacagauucucc...............................................................................................................................................................................................   26  0   LCK-3
......................uuguuccugcacagauucucc...............................................................................................................................................................................................   28  0   LT-2
......................uNguuccugcacagauucucc...............................................................................................................................................................................................   1   1   JCK-3
......................uuguuccugcacagauucucc...............................................................................................................................................................................................   28  0   LT-1
......................uuguucAugcacagauucucc...............................................................................................................................................................................................   1   1   JCK-3
......................uugNuccugcacagauucucc...............................................................................................................................................................................................   1   1   JCK-1
......................uuguuccugcacagauucucc...............................................................................................................................................................................................   13  0   JT-1
......................uuguuccugcCcagauucucc...............................................................................................................................................................................................   1   1   JCK-2
......................uuguuccugcacagCuucucc...............................................................................................................................................................................................   1   1   JCK-3
......................uuguuccugcacCgauucucc...............................................................................................................................................................................................   1   1   JCK-1
......................uuguuccugcacagauucucc...............................................................................................................................................................................................   215 0   JCK-3
.......................uguuccugcacagauucu.................................................................................................................................................................................................   1   0   LT-3
.......................uguuccugcacagauucuc................................................................................................................................................................................................   2   0   JT-2
.......................uguuccugcacagauucuc................................................................................................................................................................................................   2   0   LT-1
.......................uguuccugcacagauucuc................................................................................................................................................................................................   4   0   LCK-3
.......................uguuccugcacagauucuc................................................................................................................................................................................................   4   0   JCK-2
.......................uguuccugcacagauucuc................................................................................................................................................................................................   2   0   JT-3
.......................uguuccugcacagauucuc................................................................................................................................................................................................   3   0   LT-3
.......................uguuccugcacagauucuc................................................................................................................................................................................................   3   0   LT-2
.......................uguuccugcacagauucuc................................................................................................................................................................................................   3   0   JCK-3
.......................uguuccugcacagauucuc................................................................................................................................................................................................   4   0   JCK-1
.......................uguuccugcacagauucucc...............................................................................................................................................................................................   13  0   LCK-3
.......................uguuccugcacagauucucc...............................................................................................................................................................................................   1   0   LT-2
.......................uguuccugcacagauucucc...............................................................................................................................................................................................   12  0   JCK-2
.......................uguuccugcacagauucucc...............................................................................................................................................................................................   5   0   JCK-1
.......................uguuccugcacagauucucc...............................................................................................................................................................................................   4   0   LT-3
.......................uguuccugcacagauucucU...............................................................................................................................................................................................   1   1   LT-3
.......................uguuccugcacagauucucU...............................................................................................................................................................................................   1   1   LT-2
.......................uguuccugcacagauucucc...............................................................................................................................................................................................   5   0   JCK-3
.......................uguuccugcacagauucucU...............................................................................................................................................................................................   1   1   JCK-2
.......................uguuccugcacagauucucU...............................................................................................................................................................................................   1   1   JCK-3
.......................uNuuccugcacagauucucc...............................................................................................................................................................................................   1   1   JCK-3
.......................uguuccugcacagauucuccc..............................................................................................................................................................................................   133 0   JCK-1
.......................uguuccugcacagauucuccc..............................................................................................................................................................................................   11  0   JT-3
.......................uguuccugcacagauucuccc..............................................................................................................................................................................................   24  0   LT-1
.......................uguuccugcacagauucuccc..............................................................................................................................................................................................   5   0   JT-2
.......................uguuccugcacagauucuccc..............................................................................................................................................................................................   236 0   JCK-2
.......................uguuccugcacaAauucuccc..............................................................................................................................................................................................   1   1   JCK-2
.......................uguuccugcacagauucuccc..............................................................................................................................................................................................   13  0   LT-2
.......................uNuuccugcacagauucuccc..............................................................................................................................................................................................   1   1   JCK-3
.......................uguuccugcacagauucuccc..............................................................................................................................................................................................   171 0   JCK-3
.......................uguuccugcCcagauucuccc..............................................................................................................................................................................................   1   1   JCK-1
.......................uguuccugcacagauucuccc..............................................................................................................................................................................................   12  0   LCK-1
.......................uguuccugcacagauucuccG..............................................................................................................................................................................................   1   1   JCK-3
.......................uguuccuAcacagauucuccc..............................................................................................................................................................................................   1   1   JCK-3
.......................ugCuccugcacagauucuccc..............................................................................................................................................................................................   1   1   JCK-3
.......................uguuccugcacagauucuccc..............................................................................................................................................................................................   24  0   LT-3
.......................uguuccugcacagauucuccc..............................................................................................................................................................................................   11  0   LCK-2
.......................uguuccugcacagauucuccc..............................................................................................................................................................................................   13  0   LCK-3
.......................uguuccugAacagauucuccc..............................................................................................................................................................................................   5   1   LT-3
.......................................................acguggagauggagaaca.................................................................................................................................................................................   2   0   LT-3
.................................................................................ugggagaaucugugcaggaac....................................................................................................................................................   3   0   JCK-2
.................................................................................ugggagaaucugugcaggaac....................................................................................................................................................   2   0   JCK-3
.................................................................................ugggagaaucugugcaggaac....................................................................................................................................................   1   0   LT-3
```

Mature **Star**

nnnnnnnnnnnnnuuguugaaguuguuccugcacagauucucccauuguaauaggacguggagauggagaacaguaggaagugggagaaucugugcaggaacaacuucaacaauaucaacaagaucaacagggagcaaugggagaaaaugugcacgaacaauaucaacagaaucaacagggaagaagcacugcuccaccugguguuugccuuuccuauguaacuccaaaugccaucuuaucuauaacgca

ugggagaaucugugcaggaac	7	0	LT-2
ugggagaaucugugcaggaacaac	3	0	JT-3
ugggagaaucugugcaggaacaac	2	0	JT-2
gggagaaucugugcaggaacaac	6	0	JT-1
gggagaCucugugcaggaacaac	1	1	JT-1
gagaaucugugcaggaac	17	0	LCK-1
gagaaucugugcaggaac	3	0	JCK-1
gagaaucugugcaggaac	3	0	LT-2
gagaaucugugcaggaac	6	0	LT-3
gagaaucugugcaggaac	10	0	JCK-2
gagaaucugugcaggaac	4	0	LT-1
gagaaucugugcaggaac	11	0	LCK-2
gagaaucugugcaggaaca	7	0	JT-1
gagaaucugugcaggaaca	1	0	JCK-2
gagaaucugugcaggaaca	1	0	JCK-3
gagaaucugugcaggaacaac	2	0	LT-2
gagaaucugugcaggaacaac	11	0	LT-3
gagaaucugugcaggaacaac	10	0	JCK-1
gagaaucugugcaggaacaac	7	0	LCK-3
gagaaucugugcaggaacaac	19	0	JCK-3
gagaaucugugcaggaacaac	3	0	LT-1
gagaaucugugcaggaacaac	29	0	JCK-2
agaaucugugcaggaacaac	2	0	JCK-2
agaaucugugcaggaacaac	1	0	LT-3
agaaucugugcaggaacaac	2	0	JCK-1
agaaucugugcaggaacaac	1	0	LT-1
agaaucugugcaggaacaacu	1	0	JCK-1
agaaucugugcaggaacaacu	3	0	JT-3
agaaucugugcaggaacaacu	3	0	LT-3
agaaucugugcaggaacaacu	2	0	JCK-2
agaaucugugcaggaacaacu	4	0	LT-1
agaaucugugcaggaacaacu	4	0	LT-2
agaaucugugcaggaacaacu	2	0	JCK-3
agaaucugugcaggaacaacu	5	0	LCK-3
agaaucugugcaggaacaacu	5	0	JT-2
aaucugugcaggaaUaac	6	1	LCK-3
aaucugugcaggaacaacu	1	0	LT-1
aaucugugcaggaacaacu	1	0	LCK-3
aaucugugcaggaacaacu	1	0	JCK-2
aaucugugcaggaacaacuu	2	0	LT-1
aaucugugcaggaacaacuu	2	0	LT-3
aaucugugcCggaacaacuuc	1	1	JCK-2
aaucugugcaggaacaacuuc	39	0	JCK-2
aaucugugcaggaacaacuuc	11	0	LT-3
aaucugugcaggaacaacuuc	26	0	JCK-1
aaucugugcaggaacaacuuc	29	0	JCK-3
aaucugugcaggaacaacuuc	4	0	LCK-3
aaucugugcaggaacaacuuc	4	0	LT-1
aaucugugcaggaacaacuuca	1	0	LT-1
aaucugugcaggaacaacuuca	3	0	LT-3
aaucugugcaggaacaacuuAa	2	1	JCK-3
aaucugugcaggaacaacuuca	4	0	LCK-3
aaucugugcaggaacaacuuca	2	0	JCK-3
aaucugugcaggaacaacuuca	3	0	JCK-2
aucugugcaggaacaacu	1	0	LT-3
aucugugcaggaacaacu	1	0	JCK-2
aucugugcaggaacaacu	2	0	LT-2
aucugugcaggaacaacu	4	0	LCK-3
aucugugcaggaacaacuu	20	0	JT-1
aucugugcaggaacaacuu	1	0	LT-3
aucugugcaggaacaacuuc	21	0	JCK-3
aucugugcaggaacaacuuc	15	0	LT-1
aucugugcaggaacaacuuc	20	0	JCK-2
aucugugcaggaacaacuuc	20	0	JCK-1
aucugugcaggaacaacuuc	22	0	LT-2
aucugugcaggaacaacuuc	16	0	LT-3
aNcugugcaggaacaacuuca	1	1	JCK-3
aucugugcaggaacaacuuca	20	0	LT-3
aucugugcaggaacaacuuca	12	0	LT-1
aucugugcaggaacaacuuca	73	0	JCK-3

```
                Mature                                    Star
nnnnnnnnnnnnuuguugaaguuguuccugcacagauucucccauuguaauaggacguggagauggagaacaguaggaagugggagaaucugugcaggaacaacuucaacaauaucaacaagaucaacagggagcaaugggagaaaaugugcacgaacaauaucaacagaaucaacagggaagaagcacugcuccaccugguguuugccuuuccuauguaacuccaaaugccaucuuaucuauaacgca
.......................................................................................aucugugcaggaacaacuuca.............................................................................................................................................  19   0   LT-2
.......................................................................................aucugugcaggGacaacuuca.............................................................................................................................................  1    1   JCK-2
.......................................................................................aucugugcaggaacaacuuca.............................................................................................................................................  120  0   JCK-2
.......................................................................................aucugugcaggaacaacuuca.............................................................................................................................................  70   0   JCK-1
.......................................................................................aucugugcaggaacaacuuca.............................................................................................................................................  28   0   LCK-3
.......................................................................................aucugugUaggaacaacuuca.............................................................................................................................................  1    1   JCK-2
.......................................................................................aucugugcaggaacaacuucaac...........................................................................................................................................  1    0   JCK-2
.......................................................................................aucugugcaggaacaacuucaac...........................................................................................................................................  1    0   JCK-1
........................................................................................ucugugcaggaacaacuuc..............................................................................................................................................  2    0   LCK-3
........................................................................................ucugugcaggaacaacuuc..............................................................................................................................................  1    0   JCK-3
........................................................................................ucugugcaggaacaacuuca.............................................................................................................................................  2    0   JCK-3
........................................................................................ucugugcaggaacaacuuca.............................................................................................................................................  1    0   JCK-1
........................................................................................ucugugcaggaacaacuucaac...........................................................................................................................................  1    0   JCK-2
.........................................................................................cugugcaggaacaacuuc..............................................................................................................................................  3    0   JCK-3
.........................................................................................cugugcaggaacaacuuca.............................................................................................................................................  1    0   JCK-2
.........................................................................................cugugcaggaacaacuucaac...........................................................................................................................................  10   0   JCK-1
.........................................................................................cugugcaggaacaacuucaac...........................................................................................................................................  1    0   LT-3
.........................................................................................cugugcaggaacaacuucaac...........................................................................................................................................  3    0   LT-1
.........................................................................................cugugcaggaacaacuucaac...........................................................................................................................................  13   0   JCK-2
.........................................................................................cugugcaggaacaacuucaac...........................................................................................................................................  12   0   JCK-3
.........................................................................................cugugcaggaacaacuucaacU..........................................................................................................................................  1    1   LT-3
.........................................................................................cugugcaggaacaacuucaacU..........................................................................................................................................  1    1   JCK-2
.........................................................................................cugugcaggaacaacuucaacU..........................................................................................................................................  2    1   JCK-3
..........................................................................................ugugcaggaacaacuucaac...........................................................................................................................................  1    0   JCK-2
............................................................................................ugcaggaacaacuucaac...........................................................................................................................................  3    0   LT-1
............................................................................................ugcaggaacaacuucaacaUu........................................................................................................................................  1    1   JCK-2
............................................................................................ugcaggaacaacuucaacaauU.......................................................................................................................................  3    1   LCK-3
..............................................................................................caggaacaacuucaacaUu........................................................................................................................................  1    1   JCK-3
..............................................................................................caggaacaacuucaacaauU.......................................................................................................................................  1    1   LT-2
..............................................................................................caggaacaacuucaacaauU.......................................................................................................................................  1    1   LT-1
...............................................................................................aggaacaacuucaacaauUu......................................................................................................................................  2    1   JCK-3
...............................................................................................aggaacaacuucaacaauUu......................................................................................................................................  1    1   JCK-1
......................................................................................................................................................................cagaaucaacGgggaagaa................................................................  6    1   JT-3
......................................................................................................................................................................cagaaucaacGgggaagaa................................................................  7    1   JT-2
```
